# Metastatic sinonasal malignancies of colorectal origin: Case report and comprehensive review of the literature

**DOI:** 10.1002/ccr3.9285

**Published:** 2024-08-15

**Authors:** Andrew J. Rothka, David Goldrich, Jessyka G. Lighthall

**Affiliations:** ^1^ Penn State College of Medicine Hershey Pennsylvania USA; ^2^ Department of Otolaryngology – Head and Neck Surgery Penn State Health Hershey Pennsylvania USA

**Keywords:** Head and neck malignancy, metastatic colorectal cancer, sinonasal malignancy, sinonasal metastasis

## Abstract

Primary adenocarcinomas represent a small percentage of sinonasal malignancies. Metastasis of colorectal malignancies to the paranasal sinuses is rare, poorly understood, and typically fatal. This case documents an unusual source of metastatic sinonasal malignancy and offers comparison to a cohort of similar patients found in the literature.

## 1 INTRODUCTION

Primary adenocarcinomas of the nasal cavity and paranasal sinuses account for approximately 10%–20% of sinonasal malignancies.^1^ Metastatic malignancies to the sinonasal cavities are usually solitary and present with similar symptoms as primary sinonasal tumors.^2^ The most common malignancy that metastasizes to the nasal sinus is of salivary gland origin.^2^ The authors present a case of previously metastatic colorectal cancer presenting with new metastasis to the paranasal sinus.

## 2 CASE HISTORY/EXAMINATION

A 50‐year‐old woman with a complex history of stage IV colon cancer (status post total colectomy and multiple rounds of systemic chemotherapy) with known metastases to the liver (status post three resections) and lung (undergoing monthly systemic therapy) undergoing chemotherapy with folinic acid, fluorouracil, and irinotecan (FOLFIRI) who presented to the Emergency Department (ED) for new onset encephalopathy and generalized weakness and fatigue.

## 3 INVESTIGATIONS AND TREATMENT

**FIGURE 1:**
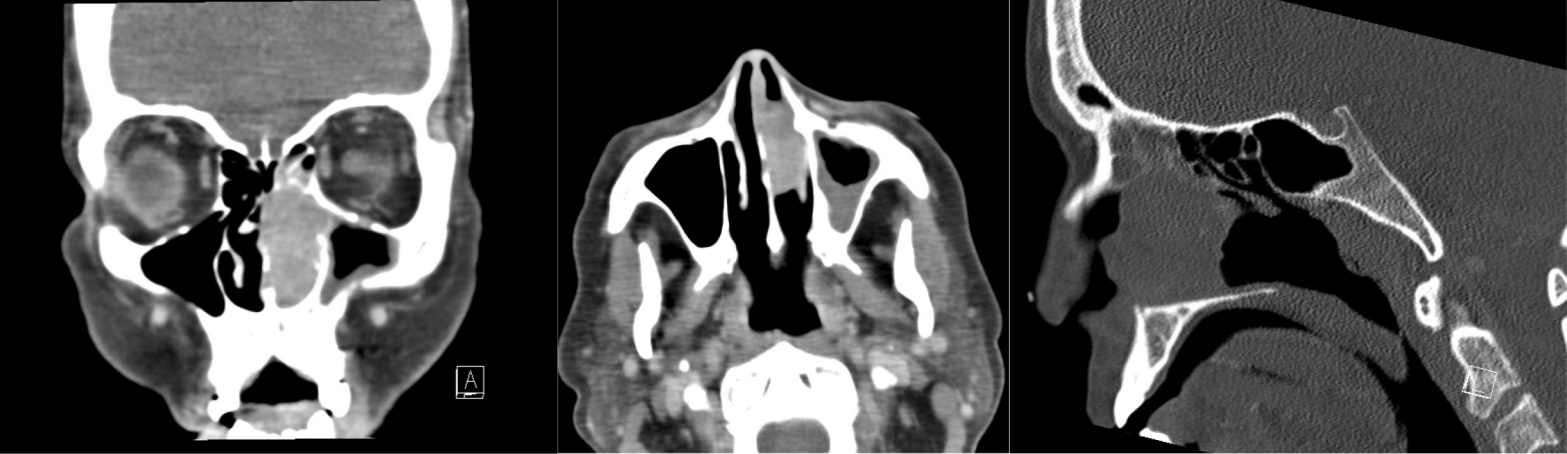
CT sinus in 3 different views of the sinonasal mass on presentation.

The patient was admitted to hematology‐oncology service for further workup. Computed tomography (CT) brain was obtained which demonstrated interval development of polypoidal mass in left nasal cavity obstructing left maxillary sinus infundibulum with associated mucosal thickening and air‐fluid level in the left maxillary sinus. Further evaluation with CT sinus confirmed left nasal cavity mass with extension into the medial superior aspect of the left maxillary sinus as well as into the inferior portion of the left ethmoid sinus (Figure 1). This finding was new compared to last head imaging from almost 2 years prior. Given new nasal mass in the setting of known metastatic colon cancer, otolaryngology was consulted for further evaluation.

On evaluation, patient was somnolent but arousable, following commands and able to answer questions. She reported having nasal obstruction for 3–4 weeks with difficulty breathing through the left naris. She also had 1 or 2 episodes of minor epistaxis on the left side. These episodes resolved with pressure and no additional treatment. She denied change in smell, postnasal drip, purulent drainage, or rhinorrhea. She had no previous history of nasal obstruction, nasal masses, nasal polyps, or sinonasal disease. On review of systems, she denied facial pain or pressure; numbness or paresis; otologic symptoms such as otorrhea, otalgia, vertigo, or aural fullness; no dyspnea, dysphagia, dysphonia, odynophagia; no bleeding, oral lesions, or purulent drainage in the mouth; and no neck masses or pain. In addition to her cancer history, her past medical history was significant for esophageal varices, depression, and multi‐drug resistant organism (MDRO) infection. Her surgical history was significant for multiple wedge resections of the liver as well as hysterectomy and bladder sling placement. She never had complications from anesthesia or bleeding. Her family history was not significant for sinonasal disease or nasal masses. She was a non‐smoker, and she denied alcohol or substance use.

Two days following otolaryngology consult, the patient underwent bilateral nasal endoscopy with a biopsy of the left nasal mass. The mass was found to originate from, and was contiguous with, the left middle turbinate. It was also hypervascular and solid in nature. It extended into the most posterior aspect of the left middle turbinate, but there was no definitive extension into the maxillary sinus. The biopsy was complicated by epistaxis that was difficult to control, but the patient was taken to recovery in stable condition. At this time, the differential diagnosis included a primary nasopharyngeal carcinoma versus further metastasis of the stage IV colon cancer.

## 4 OUTCOME AND FOLLOW‐UP

Initially, biopsy results suggested either a new primary sinonasal adenocarcinoma of the intestinal type or a metastatic carcinoma from the patient's pre‐existing colon cancer. Given that the patient already had two metastases from her primary malignancy, the metastatic carcinoma was favored. On immunohistochemistry, the mass was positive for CDX2. The patient was then begun on palliative radiotherapy to the nasal lesion in the outpatient setting following the surgical biopsy.

Four months following the surgical biopsy/debulking of the nasal mass, the patient presented to the ED with altered mental status and acute hypoxic respiratory failure and was admitted to the medical intensive care unit. Three days following her presentation to the ED, the patient succumbed to complications of her metastatic disease to her lungs, acute hypoxic respiratory failure, and septic shock.

## 5 DISCUSSION

Sinonasal malignancies tend to present with non‐specific symptoms, including nasal obstruction, epistaxis, facial pain, headache, proptosis, diplopia, impaired vision, and cranial nerve palsies.^3^ In order to determine extent of the mass, imaging modalities such as CT and positron emission tomography (PET) scans can be utilized.^4^ Performing scans of the full body can help determine if there are other metastases that have not displayed symptoms. Therefore, interdisciplinary care is crucial to managing patients with known malignancies.

Primary adenocarcinomas in the sinonasal tract can be divided into two distinct groups: salivary‐type adenocarcinomas and non‐salivary‐type adenocarcinomas. The latter group can be further classified as intestinal (ITAC) or non‐intestinal (NITAC). Biopsy and tumor markers can help distinguish between intestinal type adenocarcinoma and metastatic lesions. Treatment involves surgical resection and radiation therapy, though prognosis tends to be poor for many of these malignancies.^5^ Though some adenocarcinomas of the sinuses can resemble intestinal tissue on pathology, it is very rare for primary colorectal malignancies to metastasize to the paranasal sinuses.

According to the current literature, there are only a handful of cases reporting a metastasis of colorectal malignancy to the sinuses. “Colorectal Metastasis to Paranasal Sinus” was entered into the PubMed search engine. Articles were identified from the search, and the “Similar Articles” and “Cited By” articles were reviewed to further search for case reports on this topic. These cases are reported in Table 1.^2^, ^6^, ^7^, ^8^, ^9^, ^10^, ^11^, ^12^, ^13^, ^14^, ^15^, ^16^, ^17^, ^18^, ^19^, ^20^, ^21^, ^22^ In total, there were 20 cases of metastasis of colorectal cancer to the paranasal sinuses, including the current case report.

**TABLE 1:** Cases of colorectal cancers with sinonasal metastasis.

Author	Age of presentation	Gender	Primary tumor (stage)	Location of metastasis	Presenting symptoms	Treatment for sinonasal metastasis	Time between diagnosis and death	Cause of death	Positive immunohistochemistry findings on biopsy
Current	50	Female	Colon (IV)	Left nasal mass originating from the left middle meatus; previous metastases to lungs, mediastinal lymph nodes, and liver	Generalized weakness, fatigue, chills, encephalopathy, confusion	Palliative radiation	4 months	Acute hypoxemic respiratory failure	CDX2
bin Sabir Husin Athar et al.^6^	52	Female	Colon (N/a)	Right maxillary sinus with extension into ethmoid, frontal, and sphenoidal sinuses	Swelling and numbness of right cheek, right‐sided nasal obstruction, epistaxis, anosmia, loosening of upper teeth, weight loss, headache	Palliative radiation	3 months	Progression of disease	CK20, CEA, P53
Cama et al.^7^	57	Female	Colon (IV)	Right alveolar ridge, posterior wall of right maxillary sinus, infratemporal fossa, pterygoid muscles	Painful & gradually increasing swelling of the right alveolar ridge, right otalgia, dysphagia, painful mastication	Cannot access	Does not mention	Marked: CK20, CEA, CAM 5.2 SIMA, Ber‐EP4; Moderate: AE1/AE3, CK20	
Chang et al.^8^	70	Male	Cecum (IVc)	Right frontal sinus	Epistaxis	Tumor resection, chemotherapy	3 months	Does not mention	
Conill et al.^9^	77	Female	Rectum (T3N0)	Nasal and paranasal sinuses with extension into right orbit	Edema of the right superior eyelid with proptosis	Does not mention	Does not mention	CK20, P53	
Conill et al.^10^	85	Male	Colon (T2N0)	Left ethmoid sinus	Nasal obstruction, epistaxis, headache, hearing loss, vision loss	Palliative radiation	2 months	Complicated pneumonia with pleural empyema	CEA
Fountarlis et al.^11^	87	Male	Colon (n/a)	Internasal septum, anterior and posterior wall of frontal sinuses; Lung	3‐month history of frontal swelling	Palliative chemotherapy	Alive at time of submission	N/a	CDX2, CK20
Hwang et al.^12^	46	Female	Colon pT4N0	Nasal mass and brain metastases	Headache, vomiting	Chemotherapy followed by Palliative radiation	29 months	Progression of disease	CDX2, CK20, Villin
Hwang et al.^12^	64	Male	Colon pT3N2b	Central mass found on anterior and posterior nasoendoscopy	Epistaxis	Passed away prior to treatment of metastasis	11 months after initial diagnosis	Progressive disease and complications	Broad spectrum CK (AE1/AE3), CK20, CDX2
Kamiński et al.^13^	71	Not stated	Colon (N/A)	Sphenoid sinus	The metastasis was the first symptom of the disease	Palliative radiation	5 months	Progression of disease	Does not mention
Lahfidi et al.^14^	49	Male	Rectum	Right frontal sinus	Headache, dizziness, vomiting	Radiation	Alive at time of submission	N/a	Does not mention
Makoto et al.^15^	58	Male	Colon (IIIb)	Left maxillary sinus and gingiva; bowel recurrence, liver, lung	Swelling on left side of upper jaw and decreased oral intake, abdominal pain	Chemotherapy	12 months	Clinical deterioration due to multiple organ metastases	CEA, CA19‐9
Prakash et al.^2^	55	Female	Colon (N/a)	Left ethmoidal and sphenoidal sinuses that extended into medial wall of left orbit	Altered bowel habits for 2 months	Palliative radiation	3 months	Progressive disease and complications	CK20, CDX2, CEA
Prasad et al.^16^	51	Male	Colon (IIIB)	Posterior Right maxilla surrounding roots for first and second maxillary molars and involving the hard palate, the right greater and lesser palatine foramina, right pterygoid plates, right medial pterygoid muscle; extended into right maxillary sinus, right retromolar trigone, right retromaxillary fat, and right inferior nasal cavity mucosa; abutted anterior aspect of soft palate	Rightsided molar tooth bleeding and right‐sided palate swelling that led to difficulty speaking, eating, and a 15‐lb weight loss	Chemoradiation	Does not mention	CK20, CDX2, MUC2	
Robinson^17^	68	Female	Colon (n/a)	Right maxillary sinus	3 Weeks of painful swelling in the right cheek	Cannot access	2 months	Complications of malignancy	Does not mention
Scofield et al.^18^	68	Female	Colon (N/a)	Sphenoid wing and right ethmoid sinus; previous metastases to liver and lungs	Right upper and lower eyelid edema and erythema, decreased vision, relative afferent pupillary defect, limitation of extraocular movements, and chemosis suggestive of orbital cellulitis	Chemotherapy	6 months	Complications of malignancy	CK20, CDX2
Skalova et al.^19^	62	Male	Colon pT3N1M0	Anterior wall of maxillary sinus with orbital invasion	Swelling of maxillary area of the face with nodular skin metastatic lesions of lips, nape, and face	Does not mention	Alive at time of submission	N/a	CK20
Somali et al.^20^	47	Female	Colon (IV)	Mass involving sphenoid sinus, left maxillary sinus, left pterygopalatine plate, infratemporal fossa	Toothache and upper lip numbness	Radiation	Does not mention		
Tanaka^21^	72	Female	Rectum (N/A)	Posterior ethmoid sinus, sphenoid sinus, optic nerve	Vision loss in the right eye	Radiation	Does not mention		
Toomey et al.^22^	67	Female	Rectum (n/a)	Bilateral frontal sinuses, left anterior ethmoid sinus; lung, adrenal glands	Pain on bridge of nose radiating to both eyes	Does not mention	11 days	Bowel perforation and peritonitis	Does not mention

Abbreviations: AE1/AE3, cytokeratin AE1/AE3; Ber‐EP 4, antihuman epithelial antigen; CAM 5.2, anti‐cytokeratin; CDX2, caudal‐type homeobox 2; CEA, carcinoembryonic antigen; CK20, cytokeratin 20; MUC2, mucin 2; P53, tumor protein p53; SIMA, small intestinal mucin antigen.

The assembled cohort of patients was between 46 and 87 years old. Of the 15 cases that mention patient survival, only three were alive at time of submission of the article.^11^, ^14^, ^19^ The patient with the longest survival from diagnosis of the metastasis was 29 months.^12^ All but one patient had at least one additional metastasis elsewhere in the body when the sinonasal malignancy was discovered.^13^

Overall, the prognosis when there is a paranasal sinus metastasis is very poor. This is largely due to the insidious onset of these tumors and the start of symptomology when the lesion has become advanced.^23^ Certain occupational exposures including wood and leather dust have been associated with an increased risk of developing sinus adenocarcinoma.^24^ However, the risk factors and path of spread for colorectal malignancies in the paranasal sinuses are poorly understood. When deciding to pursue radical versus conservative therapy for such malignancies, the patient should be viewed holistically and with a multidisciplinary team to determine the best course of action for the patient.

A possible limitation for this case report includes incomplete data in the literature cohort, as each patient is reported uniquely with different information included. Additionally, the patient presented in this case did not receive magnetic resonance imaging (MRI) due to the emergent nature of the intractable bleeding. Further imaging evaluation and histopathological evaluation could provide a more complete picture of the patient presentation.

## 6 CONCLUSION

Metastasis of malignancy of colorectal origin to the head and neck is rare and typically associated with a poor overall prognosis. Identifying signs of malignancy are important on physical exam, and a thorough past medical history can help identify if there is a suspicion of an unusual origin of a malignancy in the head and neck. Biopsy with immunohistochemistry can suggest the diagnosis; however, this may require evaluation in the context of the overall patient picture.

## AUTHOR CONTRIBUTIONS

**Andrew J. Rothka:** Conceptualization; investigation; writing – original draft; writing – review and editing. **David Goldrich:** Conceptualization; investigation; writing – original draft; writing – review and editing. **Jessyka G. Lighthall:** Conceptualization; investigation; project administration; writing – original draft; writing – review and editing.

## FUNDING INFORMATION

There were no sources of funding for this case report.

## CONSENT

Written informed consent was obtained from the patient to publish this report in accordance with the journal's patient consent policy. All protected health information was withheld from this piece to ensure patient anonymity.

## Data Availability

Data sharing is not applicable to this article as no datasets were generated or analyzed during the current study.
